# Potential Application of Curcumin and Its Analogues in the Treatment Strategy of Patients with Primary Epithelial Ovarian Cancer

**DOI:** 10.3390/ijms151221703

**Published:** 2014-11-25

**Authors:** Katarzyna M. Terlikowska, Anna M. Witkowska, Malgorzata E. Zujko, Bozena Dobrzycka, Slawomir J. Terlikowski

**Affiliations:** 1 Department of Food Science and Technology, Medical University of Bialystok, Szpitalna 37, 15-295 Bialystok, Poland; E-Mails: witam@umb.edu.pl (A.M.W.); malgorzata.zujko@umb.edu.pl (M.E.Z.); 2 Department of Obstetrics, Gynaecology and Maternity Care, Medical University of Bialystok, Warszawska 15, 15-062 Bialystok, Poland; E-Mails: bdobrzycka@gmail.com (B.D.); sterlikowski@gmail.com (S.J.T.)

**Keywords:** ovarian cancer, chemoprevention, target therapy, curcumin, curcumin analogues

## Abstract

Recent findings on the molecular basis of ovarian cancer development and progression create new opportunities to develop anticancer medications that would affect specific metabolic pathways and decrease side systemic toxicity of conventional treatment. Among new possibilities for cancer chemoprevention, much attention is paid to curcumin—A broad-spectrum anticancer polyphenolic derivative extracted from the rhizome of *Curcuma longa* L. According to ClinicalTrials.gov at present there are no running pilot studies, which could assess possible therapeutic benefits from curcumin supplementation to patients with primary epithelial ovarian cancer. Therefore, the goal of this review was to evaluate potential preclinical properties of curcumin and its new analogues on the basis of *in vivo* and *in vitro* ovarian cancer studies. Curcumin and its different formulations have been shown to display multifunctional mechanisms of anticancer activity, not only in platinum-resistant primary epithelial ovarian cancer, but also in multidrug resistant cancer cells/xenografts models. Curcumin administered together with platinum-taxane chemotherapeutics have been reported to demonstrate synergistic effects, sensitize resistant cells to drugs, and decrease their biologically effective doses. An accumulating body of evidence suggests that curcumin, due to its long-term safety and an excellent profile of side effects should be considered as a beneficial support in ovarian cancer treatment strategies, especially in patients with platinum-resistant primary epithelial recurrent ovarian cancer or multidrug resistant disease. Although the prospect of curcumin and its formulations as anticancer agents in ovarian cancer treatment strategy appears to be challenging, and at the same time promising, there is a further need to evaluate its effectiveness in clinical studies.

## 1. Introduction

Primary epithelial ovarian cancer is the leading cause of death from gynaecological diseases and the fifth cause of cancer deaths among women worldwide [1]. Non-specific symptoms in the initial stage of the disease, lack of valuable screening tests and a non-defined group of women who should be subjected to such a program, result in as much as 70% of epithelial ovarian cancers being diagnosed at the advanced stage [2]. Despite early response in 70%–80% patients undergoing conventional treatment based on an aggressive surgical cytoreduction as well as adjuvant platinum or taxane chemotherapy, many patients relapse and ultimately die because of platinum-sensitive, platinum-resistant or multidrug resistant disease [3]. According to the Gynecologic Cancer Intergroup (GCIG) criteria, relapse may be predicted several months before clinical or radiological symptoms by measurement of cancer antigen CA125, but as reported, early institution of second-line chemotherapy does not affect general survival [4]. Therefore, it is crucial to develop effective drugs that will improve life expectancy as well as decrease systemic side effect toxicity.

### Biological Response Modifiers in Systemic Therapy of Ovarian Cancer

Target therapies include small molecules and monoclonal antibodies which can inhibit or induce mechanisms involved in regulation of carcinogenesis. So far, the most extensively investigated targeted therapies were focused on inhibition of angiogenesis and metastasis. So called “VEGFR (vascular endothelial growth factors receptors) tyrosine kinase inhibitors (TKIs)” such as cediranib and sorafenib, or humanized monoclonal IgG1 antibodies were tested against vascular endothelial growth factors family (VEGF) members [5,6]. An angiogenesis inhibitor, Bevacizumab, creates a complex with VEGF and thus inhibits the action of the VEGF ligand involved in neovascularization [7,8]. In third phase clinical trials of gynecologic oncology group (GOG 218) and gynecologic cancer intergroup (ICON-7), statistically significant increases in the average time of progression-free survival were observed after a single-agent drug administration or bevacizumab plus carboplatin-taxane treatments, but no improvement in the overall survival rates were demonstrated [9,10,11]. It is worth noting that bevacizumab is the sole anti-angiogenic drug approved in Europe, which at a dose of 7.5–15 mg/kg can be used in front-line therapy in combination with platinum-based drugs for ovarian epithelial cancer treatment [12]. Since there are concerns about the high rate of intestinal perforation and nervous system toxicity seen in some studies, patients for this therapy should be carefully selected [11,13].

Ovarian cancers with mutated *BRCA1/2* genes are particularly sensitive to agents that cause DNA double strand breaks (DSBs) and DNA interstrand cross-links, such as platinum compounds and poly (ADP-ribose) polymerase (PARP) enzyme inhibitors, for example olaparib and iniparib [14,15]. Olaparib seems to be the most clinically advanced PARP inhibitor for ovarian cancer treatment. The most frequently reported causally related adverse events in second phase clinical trial with orally active olaparib in women with confirmed genetic *BRCA1/2* mutation were of a rather low toxicity scale, e.g. nausea, fatigue and anemia [16]. However, in combination with chemotherapeutic agents, olaparib increased bone marrow toxicity to levels greater than observed with the drug alone [17]. In another second phase study, significant associations between the clinical benefit rate and platinum-free interval which included platinum-sensitive, resistant and refractory subgroups after olaparib treatment (69%, 45%, and 23%, respectively) were observed [18].

As most tumors have an excessive growth rate and their demand for folate increases, great attention was being paid toward drugs that inhibit folate metabolism in cancer cells, such as farletuzumab (humanized mAb), thymidylate synthase inhibitor BGC945 or pemetrexed [19,20,21]. Best results were observed for vintafolide, which acts directly against the folate receptor FR-α [22]. Vintafolide (EC145) is a small molecule drug conjugate, consisting of folic acid linked to the vinca alkaloid desacetylvinblastine hydrazine, which is a potent microtubule destabilizing agent [23].

For successful treatments in patients with resistant disease, drugs could be selected on the basis of response to predictive biomarkers e.g., an extracellular domain of CA125 (also known as MUC16). It was shown that the *C*-terminal domain of MUC16 promotes cisplatin resistance and selectively modulates the sensitivity of epithelial ovarian cancer (EOC) cells to DNA-damaging drugs such as cyclophamide, doxorubicin and etoposide [24]. In preclinical and clinical studies, antibodies and vaccines directed against mucins have influenced on higher survival and delayed metastasis [25,26,27].

Considering given examples, it is becoming increasingly evident that drugs which inhibit selected molecular targets and multiple signaling pathways such as VEGFR, EGFR, IL-6R-JAK-STAT3/Nf-κB/PI3K/AKT/mTOR may enhance standard chemotherapy or even act independently in both platinum-sensitive and resistant disease in order to prolong progression-free survival and overall survival rates [28]. Current standard therapies do not achieve satisfactory results especially in patients with cisplatin or multidrug resistant disease. What is more, a significant decrease in quality of life due to severe side effects of treatment has been observed. Currently, there are no United States Food and Drug Administration Service (FDA) approvals of any targeted agents in ovarian cancer treatment (studies are in IId or IIId phase). In line with this, a review of the natural anticancer properties of curcumin and its analogues was evaluated in terms of their utility for ovarian cancer treatment strategy.

## 2. Curcumin—General Description and Pharmacokinetics

Turmeric (*Curcuma longa* L.) also called Indian curcuma belongs to the ginger family. This plant grows in a hot subtropical climate predominantly in Asia, mainly India and China. Turmeric—A yellow orange powder obtained from rhizome is being used for centuries as a dye in the textile industry, ingredient of different spice mixes as well as a healing substance [29]. The rhizome of turmeric comprises approximately 70% starch, 3%–5% curcuminoids and volatile oils [30,31]. The main component of turmeric which provides healing effect is curcumin. This natural polyphenolic plant pigment has been shown to display multiple functions such as antioxidant, antibacterial, anti-inflammatory, analgesic and wound-healing properties [32,33,34,35]. Additionally, curcumin has been reported as anticarcinogenic substance, which induces apoptosis and can inhibit angiogenesis as well as tumor metastasis [11,36,37].

In 2004, The Joint FAO/WHO Expert Committee on Food Additives (JECFA) allocated an Adequate Daily Intake (ADI) of curcumin of 0–3 mg/kg bw/day [38]. Nevertheless, short-term studies in humans indicated that curcumin did not cause toxic and adverse effects at a dose of 8 g per day [39,40]. In about 30% of the participants supplemented with 12 g of curcumin per day adverse effects were diarrhea, rash, headache and yellow discolouration of faeces (first degree in a scale of toxicity according to the National Cancer Institute) [40]. The oral lethal doses of curcumin were estimated in mice and rats on 2–10 and 5–10 g/kg bw/day, respectively [38].

The main problematic issue connected to limited application of curcumin as an anticancer agent is its poor bioavailability. Pharmacokinetics of curcumin is now being extensively investigated. By now it appears that curcumin has a hydrofobic nature and in the organism it is converted to more water-soluble form via conjugation reaction. Concentration of curcumin in peripheral veins seems to be small due to its biotransformation, reduction and conjugation with glucuronic acid and sulfate by cytochrome P450 (CYP) enzymes located in the gut epithelium or in the liver [41]. Administration of 4, 6 and 8 g of curcumin per day for 3 months in patients with precancerous lesions resulted in mean curcumin plasma concentrations ranging from 0.51 and 0.63 to 1.77 μM respectively, 1–2 h post dose [42]. In this study urinary excretion of curcumin proved to be undetectable [42]. However, in patients with advanced colorectal cancer dosed with 3.6 g of curcuminoids (450 mg curcumin, 40 mg demetoxycurcumin and 10 mg bisdemetoxycurcumin) per day up to 3 months, mean plasma concentrations 1 h after administration were 8.9 and 15.8 nmol/L for curcumin sulfate and curcumin glucuronide, respectively [43]. In addition, urinary levels of curcumin, curcumin sulfate and curcumin glucuronide varied between 0.1–1.3 µmol, 19–45 nmol/L, 210–510 nmol/L, respectively. The most abundant detected curcuminoid in feces was curcumin (25–116 nmol/g dried feces) [43]. The presence of curcumin conjugates in blood and curcumin in secretions explains low circulating levels of free curcumin. Generally, it is assumed that blood concentration of so called “curcumin” and subsequently its anticancer properties depend on types of isomeric compounds supplemented (demetoxy-, bisdemetoxy-, and tetrahydroxycurcumin), duration of use, frequency and size of daily-administered dose, and forms of curcumin delivery system (e.g., gel capsules, micelles). It was shown that tetrahydroxycurcumin is a less potent inhibitor of nuclear factor κβ (NFκβ), cyclooxygenase-1 (COX-1), 5-lipoxygenase (5-LOX) and cell proliferation at equivalent concentration as curcumin, demetoxy- or bisdemetoxycurcumin [44,45]. Some attempts to improve the solubility of hydrophobic curcumin and thereby to increase its bioavailability have been made. For example, curcumin can be complexed with metal ions (Zn^2+^, Cu^2+^, Mg^2+^, Se^2+^) and albumin. The bioavailability of curcumin can also be enhanced by addition of piperine, phospholipids, or by encapsulation in liposomes [30]. Structurally modified analogues of curcumin unconjugated or conjugated to a ligand or antibody enable the drug to target specific receptors or epitopes on the surface of cancer cells and thus provide not only prophylactic but also therapeutic properties of curcumin [46,47,48].

### 2.1. Anticancer Properties of Curcumin

**Figure 1:**
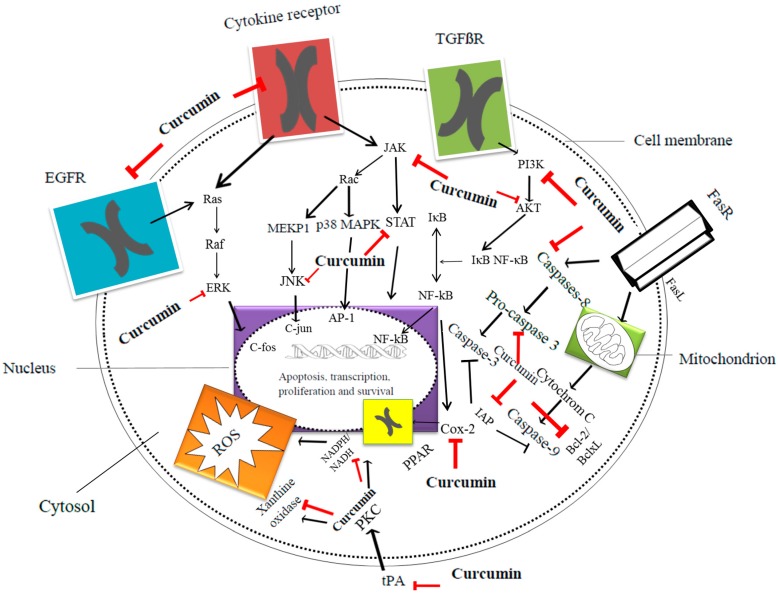
Major apoptotic and inflammatory pathways regulated by curcumin [49]. Courtesy of Postepy Higieny i Medycyny Doswiadczalnej. AKT: Serine/threonine-specific protein kinase; AP-1: Activator protein 1, transcriptional factor; Bcl-2: B-cell lymphoma 2, regulator protein; BclxL: B-cell lymphoma-extra large antiapoptotic protein; c-*fos*: c-*fos* protein, proto-oncogen; c-*jun*: c-*jun* protein, proto-oncogen; COX-2: Cyclooxygenase-2; EGFR: Epidermal growth factor receptor; ERK: Extracellular-signal-regulated kinases; FasR: Death receptor; FasL: Type-II transmembrane protein; IAP: Inhibitor of apoptosis protein family; IκB: Inhibitor of κB; JAK: Janus kinase; JNK: c-*jun N*-terminal kinases; MEKP1: Mitogen-activated protein kinase 1; NADH: Reduced nicotinamide adenine dinucleotide; NADPH: Reduced nicotinamide adenine dinucleotide phosphate; NFκB: Nuclear factor κ-light-chain-enhancer of activated B cells; p38 MAPK: p38 mitogen-activated protein kinases; PI3K: Phosphatidylinositol-4,5-bisphosphate 3-kinase; PKC: Protein kinase C; PPAR: peroxisome proliferator-activated receptor; Rac: Subfamily of the Rho family of GTPases; Raf: Raf family kinases; Ras: Ras family kinases; ROS: Reactive oxygen species; STAT: Signal transducer and activator of transcription protein family; TGFβR: Transforming growth factor β receptor; tPA: Tissue plasminogen activator; Black arrows represents signaling pathways, while red symbols, structures inhibited via curcumin.

Cell signaling pathways mediated by curcumin are shown in Figure 1 [49]. Curcumin anticancer effects in epithelial ovarian cancer cultures are shown in Table 1 [50,51,52,53,54,55,56,57,58,59,60].

**Table 1:** Pleiotropic mechanisms of curcumin action in epithelial ovarian cancer cell/xenograft models. ↑ (high expression), ↓ (low expression).

Curcumin Concentration	Cancer Cell Line	Assessed Parameter	Outcome Measures	Reference
50 μM	SKOV3	↓MMP-9, ↓CD44, ↓osteopontin	↓Invasion of SKOV3 cells	[50]
40 μM	HO-8910	↓Bcl-2, ↓Bcl-xL, ↓pro caspase-3, ↑ p53, ↑Bax	↓Cell growth, ↑apoptosis	[51]
10–50 μM	A2780	↓Bcl-2, ↓p53, no changes in MDM2, ↓NFκB, ↑caspase-3	↓Cell growth, ↑apoptosis	[52,53]
10–50 μM	CaOV3	↑AMPK, ↑p38, ↑p53 phosphorylation	↓Proliferation, ↑apoptosis	[54]
0.1–100 μM	2008, C13	↑ROS, ↓glutathione	↓Cell proliferation, ↑apoptosis with curcumin alone, synergistic effect with cisplatin or oxaliplatin, ↓cell cycle via synergistic effect with cisplatin or oxaliplatin, ↑sensitivity to cisplatin in resistant C13 cells	[55]
40 μM	CaOV3	↓AQP-3	↓EGF-induced cell migration	[56]
3.12–50 μM	OVCA420, OVCA429	↑Caspase-3, ↓IL-6, ↓STAT-3 phosphorylation, ↓p-JAK-1 and p-JAK-2, ↓PIAS-3, SOCS-3	↓Cells growth, ↑apoptosis	[57]
2–80 μM	HEY, OVCA429, OCC1, SKOV3	↓Procaspase-3, ↑active caspase-3, ↓PARP-1 substrate, ↑cytochrome c, ↓Bcl-2, surviving, ↓PI3K/Akt pathway, ↑p38 MAPK pathway	↑Apoptosis: ↓Cell densities, marked cell rounding, long cytoplasmic projections, membrane blebs, DNA fragmentation	[58]
10–60 μM	SKOV3	↑*miR-9*, ↓phosphorylation of AKT and FOXO1	↓Cell proliferation, ↑apoptosis, ↓ cells growth	[59]
5–10 μM	PA-1 OVCAR-3	↓LPA-induced STAT3 phosphorylation	↓LPA-induced IL-6 and IL-8 production, ↓cell motility	[60]

MMP-9: Matrix metalloproteinase 9; CDD44: Cell surface antigen 44; Bcl-2: B-cell lymphoma 2, regulator protein; BclxL: B-cell lymphoma-extra large antiapoptotic protein; p53: Tumor protein; Bax: Apoptosis regulator protein; MDM2: Mouse double minute 2 homolog, proto-oncogene; AMPK: 5' AMP-activated protein kinase; p38: p38 MAPK mitogen-activated protein kinases; ROS: Reactive oxygen species; AQP-3: Aquaporin water channel 3; IL-6,-8: Interleukin-6,-8; STAT-3: Signal transducer and activator of transcription protein 3; p-JAK-1/JAK-2: Phosphorylated janus kinase 1, 2; PIAS-3: Protein inhibitor of activated STAT-3; SOCS-3: Suppressors of cytokine signaling protein 3; PARP-1: Poly (ADP-ribose) polymerase 1; PI3K: Phosphatidylinositol-4,5-bisphosphate 3-kinase; *miR-9*: microRNA gene; Akt: Serine/threonine-specific protein kinase; FOXO1: Forkhead box protein O1, transcription factor; LPA: Lysophosphatidic acid.

### 2.2. Curcumin Analogues in Ovarian Cancer Target Treatment

**Figure 2:**
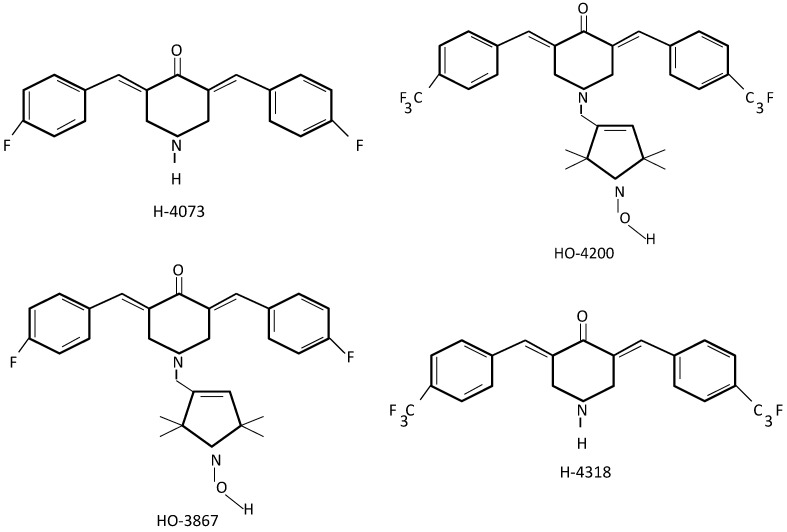
Structures of diarylidenyl piperidones (DAPs) [63].

As for now, the most extensively investigated analogues of curcumin in ovarian cancer treatment seem to be diarylidenyl piperidones (DAPs) [61]. DAPs have been synthesized by shortening and incorporation of a piperidone ring within the β-diketone backbone structure of curcumin, and by additional fluorination of the phenyl groups [62]. Several DAPs (H-4073, HO-3867, H-4318 and HO-4200) have been designed to evaluate anticancer properties *in vitro* in human cancerous (breast, colon, head, neck, liver, lung, ovarian, prostate cancer) and healthy cells (smooth muscle, aortic endothelial and epithelial ovarian surface cells) Figure 2 [63]. These analogues demonstrate substantially higher antiproliferative activity and are more effective for induction of apoptosis than curcumin alone [64]. Interestingly, DAPs in general were more preferentially cytotoxic to ovarian (A2780) and colon cancer (HCT-116) cell lines rather than lung or prostate [63]. Recent research on the curcumin analog HO-3867 containing *N*-hydroxypyrroline group has demonstrated less toxic effects to non-cancerous cells [63,64,65,66,67,68]. A number of studies were also performed on various types of ovarian cancer cell lines A2780, A2780cDDP, OV-4, SKOV3, PA-1, and OVCAR-3 and also in a murine xenograft tumor (A2780) model. It was shown that under *in vitro* and *in vivo* conditions, HO-3867 influenced apoptotic pathways by decreasing levels of mitochondrial proteins Bcl-xL, Bcl-2 and survivin, as well as by lowering VEGF expression in all cancer cell lines. This caused disruption in the JAK/STAT3 signaling pathway and in consequence led to apoptosis [69]. In addition, HO-3867 inhibited growth of the ovarian xenograft tumor (A2780) in a dose-dependent manner with slight loss of viability in healthy cells and no apparent effect on body weight. The tumor weight after dosage of 50 and 100 ppm of HO-3867 administrated orally was 0.6 and 0.2 g respectively, compared to 1.2 g untreated controls [69]. HO-3867 affected on up-regulation of tumor protein p53, p21 and decreased levels of cyclin dependent kinase 2 (cdk2) and cyclin-A which partially arrested G2/M cell-cycle and in consequence led to caspase-8 and caspase-3 activation and ultimately to apoptosis [69]. In a study using A2780 and SKOV3 cancer cell lines, HO-3867 was shown to inhibit migration and invasion of the carcinoma cells through the FAS/FAK pathway under *in vitro* and *in vivo* conditions [69]. Interestingly, in both lines, HO-3867 blocked FAS/FAK regulating genes: pHER2, pERK1/2, SREBP1 and proteins MMP-2, VEGF through accelerated ubiquitin-dependent degradation caused by decreased levels of isopeptidase USP2a [69]. Moreover, the cellular activity of HO-3867 was substantial also to cisplatin-resistant ovarian cancer cell line A2780R [70]. It was demonstrated that HO-3867 can sensitize cisplatin-resistant ovarian carcinoma through STAT3 inhibition [70]. No significant effect on cell viability after 10 µg/mL of conventional chemotherapeutic treatment based on platinum compounds (cDDP) in A2780R was observed. However, administration of cDDP with HO-4073 or HO-3867 at a dose of 5 and 10 μM exhibited a concentration-dependent cytotoxic activity with significant effect [71]. Major cytotoxic effect of DAP’s as a single agent was observed at a concentration of 10 μM [66]. Cellular uptake of 10 μM HO-3867 was over 10-fold greater than 100 μM curcumin in A2780 and what is more HO-3867 entered into cells more rapidly and its unchanged version was retained longer than curcumin [64].

**Figure 3:**
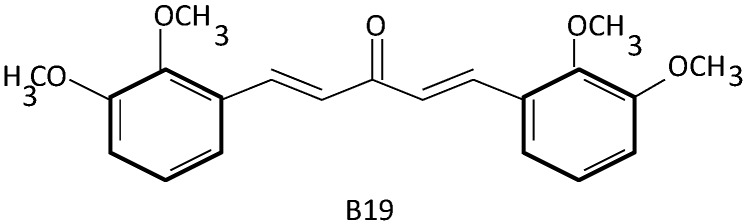
Structure of a mono-carbonyl analogue of curcumin [74].

Similar observations concerning decreased levels of Bcl-xL and Mcl-1 were observed in human ovarian carcinoma cells A2780CP treated *in vitro* with nanocapsulated curcumin (nano-CUR) [71,72]. This small molecule consisting of curcumin conjugated to monoclonal antibody specific for ovarian cancer cells (anti-TAG-72 mAb, CC49) was encapsulated in a biodegradable, bioavailability-enhancing PLGA poly-(lactic-co-glycolic-acid) layer [72]. The 6 hours-application of nano-CUR substantially decreased proliferative and cloning ability of cisplatin resistant cells and thus enabled reduction of the dosage of cisplatin and radiation required for treatment [72].

(1*E*,4*E*)-1,5-bis(2-Methoxyphenyl)penta-1,4-dien-3-one (Figure 3), a mono-carbonyl analogue of curcumin, known as B19, has been demonstrated to: cause endoplasmic reticulum (ER) stress-related autophagy, produce reactive oxygen species and initiate apoptosis in epithelial ovarian cancer HO8910, A2780, CP70 cell lines [73,74]. The cells treated with B19 were shown to have elevated levels of autophagy inhibitor 3-methyladenine (3-MA). Treated cells promoted formation of ubiquitinated misfolded proteins and induced ER stress response [73]. The accumulation of misfolded proteins in the ER, activated the unfolded protein response (UPR) and ultimately led to autophagy [73,74,75,76].

**Figure 4:**
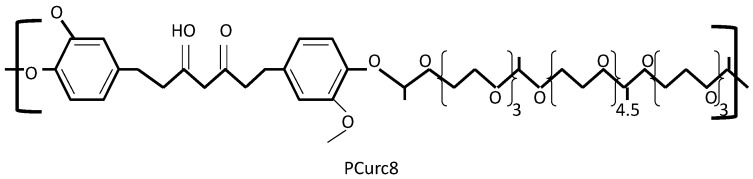
Structure of a polycurcumin PCurc8 [77].

Polycurcumin (PCurc8) (Figure 4), synthesized by polycondensation and polymerization with polyethylene glycol 200 and divinyl ether, has been shown to be highly cytotoxic to SKOV3 and OVCAR-3 cancer cells. PCurc8 IC_50_ values needed for induction of therapeutic effect were much lower if compared to curcumin (1.2 and 0.4 *vs.* 3.8 and 1.1 μg/mL respectively) [77]. Cytotoxic effect of PCurc8 was achieved by down regulation of cyclin CD1/CDK4, CDK6 and promotion of conversation of pro-caspase-3 to caspase-3. Activity of PCurc8 was also observed against SKOV-3 xenograft tumor athymic nude mice model. At a dose of 100 mg/kg PCurc8, a 68% decrease in tumor growth was observed and, what is more, significant losses in average tumor weights in treated and control mice were observed [77].

### 2.3. Curcumin and Its Analogues in Ovarian Cancer Drug Resistance

The mechanism underling intrinsic and acquired development of tumor resistance has attracted much attention. Factors associated with resistance include those which influence genomic stability through homologous recombination repair and promotion of DNA interstrand cross-links. Some mechanisms involved in this process include increased cell surface expression of the ABC superfamily of membrane transporters [78], decreased expression of the mismatch repair proteins 1 and 2 MSH2, MLH1 [79] or increased expansion of the variant polymerase, pol β [80,81]. High levels of DNA excision repair (ERCC1) protein [82], mutations or down-regulation of MLH1, MSH2 and MSH1 [83], and secondary mutations of *BRCA1/2* genes [84] have been shown to be involved in platinum-resistant ovarian cancer development. Recent studies indicate that acquired cisplatin resistance in ovarian tumors may by partially caused by reactivation of the Fanconi anemia (FA)/BRCA pathway [85]. FA is a rare autosomal recessive disease that in children causes pancytopaenia and bone-marrow failure [86]. Elder patients who survive bone-marrow disease at the age of 30 are predisposed to acute myelodysplastic leukemia, squamous cell carcinomas of the head and neck or gynaecological system [87]. Recent study has demonstrated that BRCA1 protein is also a critical component of the Fanconi pathway and that BRCA1 may itself be a Fanconi anemia gene [88]. This information is of importance for patients at high risk of hereditary breast and ovarian cancer (HBOC) development, since in HBOC, mutation in BRCA1/2 genes is frequent. Curcumin has been shown to sensitize ovarian and breast carcinoma cells to cisplatin by inhibition of the FA/BRCA pathway in a dose-dependent manner (3–20 μmol/L) [89]. In cisplatin-treated cells curcumin down-regulated the FA/BRCA pathway via 30% reduction of monoubiquitynated long form of Fanconi anemia group D2 protein (FANCD2) [89]. However, it was shown that curcumin seems to specifically sensitize cisplatin-mediated DNA damage rather than microtubular damage caused by paclitaxel [89]. These results suggest that the FA/BRCA pathway does not play a role in cellular sensitivity to paclitaxel [89]. One of the major mechanisms of tumor acquired multiple drug resistance (MDR) is the enhanced ability of drug-resistant cells to actively efflux drugs, which in consequence do not provide their toxic effects [90]. In classical way, active drug efflux is mediated via elevated expression of P-glycoprotein transporter (P-gp), mitoxantrone resistance protein (MXR) and multidrug resistance protein I (MRP1) [90]. Curcumin has been shown to overcome MDR [90,91]. Combination of curcumin and paclitaxel encapsulated in flaxseed oil containing nanoemulsion formulations led to improvement of limited intestinal absorption of chemotherapeutic and significantly decreased its IC_50_ values in wild-type SKOV3 and drug resistant SKOV3 (TR) human ovarian adenocarcinoma cells [92]. These effects were achived due to inhibition of P-glycoprotein transporter and inhibition of NFκβ [92]. In mice bearing MDR ovarian cancer HeyA8-MDR xenograft tumors, treatment with curcumin alone or in combination with docetaxel resulted in significant 47% and 58% reductions in tumor growth [93]. Curcumin and paclitaxel administered in nanoemulsion to SKOV3 tumor-bearing immunodeficient nu/nu mice at a dose of 50 mg/kg for 3 consecutive days resulted in down-regulation of intestinal P-glycoprotein (P-gp) and cytochrome P450 3A2 (CYP3A2) protein levels [94].

Curcumin has been shown to sensitize cisplatin-resistant ovarian cancer cell lines via inhibition of proliferation and activation of apoptosis [95]. By arresting G2/M phase, curcumin enhanced p53 phosphorylation and activated caspase-3 cascade followed by PAPR degradation [95]. Curcumin also enhanced phosphorylation of p38 mitogen-activated protein kinases (p38 MAPK) and inhibited phosphorylation of AKT [96]. In addition, some reports have indicated that curcumin could sensitize ovarian cancer cells CAOV3 and SKOV3 to cisplatin by inhibition of autologous production of IL-6 [57]. Moreover, curcumin and its derivatives showed synergistic drug effects on resistant ovarian cells not only with cisplatin [97,98] but also with oxaliplatin [99]. Best results were observed when oxaliplatin was administrated 4 h before curcumin dosage [99].

### 2.4. Curcumin and Clinical Studies

Though studies on curcumin and its analogues have not yet fully overcome animal models, there is some clinical evidence of its beneficial anticancer support in humans. For example, in IId phase clinical trial, 25 patients with advanced pancreatic cancer received capsules containing 1 g of curcuminoids at a total dose of 8 g/day for 8 weeks [100]. In this study curcumin down-regulated expression of NFκβ, COX-2 and pSTAT3 in peripheral blood mononuclear cells from the majority of enrolled patients [100]. Combination of standard administration of docetaxel (100 mg/m^2^) and 8 g/day of curcumin for seven consecutive d every 3 weeks in patients with advanced and metastatic breast cancer resulted in 30% and 21% decreases in VEGF tissue expression during third and sixth treatment cycle, however the data do not explain whether these values correspond to the curcumin level or the type of response to the combination therapy [101]. What is more, in this study, levels of CA15.3 remained constant after curcumin and docetaxel courses, whereas Carcinoembryonic Antigen CEA levels decreased significantly from the third cycle of the treatment [101]. Some studies show that curcumin is well-tolerated by normal tissues. Patients with colorectal cancer who received 1.8 and 3.6 g of curcumin daily within a week before surgery had higher mean values of curcumin glucuronide and curcumin sulfate in normal mucosa compared to malignant colorectal tissues (19.6 *vs.* 6.7, 12.7 *vs.* 7.7 nmol/g, respectively) [102]. Notably, higher doses of curcumin do not result in higher concentrations in tissues. The data provided from these and other pilot studies are in agreement that curcumin is well-tolerated and safe without a tendency to accumulate, and its potent chemotherapeutic action occurs in tumor response, disease stabilization or even disease regression [100,101,102,103,104]. Powerful pleiotropic properties of curcumin are mainly, but not only, caused by functional or genomic inhibition of enzymes generating ROS or inflammatory lipids (COX, LOX, xanthine oxidase, NOs), inhibition of pro-inflammatory transcription factors (NFκβ, STAT3), kinases (PKC, EGFR, tyrosine kinase) and up-regulation of anti-oxidant pathways mediated via activation of Nrf2 [105]. The idea of curcumin as chemotherapeutic agent is rather optimistic and some current studies are focused on enhancement of curcumin bioavailability and evaluation of its efficiency in different malignancies (Table 2).

**Table 2:** Summary of clinical trials scheduled for curcumin assessment as an element of anticancer strategies (according to ClinicalTrials.gov.).

Cancer	Inclusion Criteria	Intervention	Outcome Measures	Phase	Clinical Trial Number
Endometrial carcinoma	Recurrent with no life-threatening metastases	Curcuphyt (curcumin analogue), standard chemotherapy	Anti-inflammatory effect	Recruiting, 2	NCT02017353
Prostate cancer	Life expectancy > 5 years	Curcumin, curcumin analogue BCM95, radiotherapy	Radiosensitizing and radioprotective effect	Recruiting, data not shown	NCT01917890
Breast cancer	Completed chemotherapy	Curcumin, radiotherapy	Level of NF-κβ DNA binding	Not yet recruiting, 2	NCT01740323
Colorectal cancer	Familial adenomatous polyposis, stage 0	Curcumin	Number and size of polyps, side effect of curcumin, involved pathways	Recruiting	NCT00641147
Lymphocytic lymphoma Lymphocytic leukemia	Stage 0,1,2	Curcumin, vitamin D	Overall survival response, Overall survival rates, progression free survival	Not yet recruiting, 2	NCT02100423
Prostate cancer	Metastatic cancer, castration resistant	Curcumin, Taxotere	Time to progression, tumor response by RECIST criteria	Recruiting, 2	NCT02095717
Colorectal cancer	Metastatic cancer	Curcumin, chemotherapy	Neuropathic side-effect, disease response, disease survival, level of biomarkers	Recruiting, 1, 2	NCT01490996
Colon cancer	First diagnosed primary tumor without any treatment	Curcumin, curcumin conjugated with plant exosomes (Exo-cur)	Efficiency of plant exosomes in delivering curcumin to normal colon tissue and colon tumor	Recruiting, 1	NCT01294072
Intestinal adenomas	Familial adenomatous polyposis with an intact colon or with surgery	Curcumin (Calcumin)	Regression of intestinal adenomas	Recruiting, data not shown	NCT00927485
Solid tumors	Advanced or metastatic cancer, life expectancy > 3 months	Liposomeal curcumin intravenous	Safety, tolerability and pharmacokinetic of liposomeal curcumin, tumor response by RECIST criteria	Recruiting, 1	NCT02138955
Breast cancer	Atypical ductal breast hyperplasia *BRCA1* gene mutation *BRCA2* gene mutation ductal breast carcinoma *in situ* lobular breast carcinoma *in situ*	Nanoemulsion formulation of curcumin	Adherence, tolerability and safety of curcumin, anti-inflammatory changes	Recruiting, pilot study	NCT01975363
Colorectal cancer	Metastatic cancer	Curcumin, irinotecan	Safety, pharmacokinetics and effectiveness of irinotecan in combination with curcumin	Recruiting, 1	NCT01859858
Colorectal cancer	Familial adenomatous polyposis, stage 0	Phospholipid curcumin, anthocyanin extract	Markers, apoptosis, cell proliferation	Recruiting, 2	NCT01948661
Prostate cancer	Stage T1–T3	Curcumin, curcumin analogue BCM-95CG	Time of recurrence-free survival	Recruiting, 2	NCT02064673

## 3. Conclusions

Curcumin analogues presented in this review demonstrate anticancer potential not only in platinum-resistant primary epithelial ovarian cancer but also in multidrug resistant cancer cell/xenograft models. Curcumin analogues together with platinum-taxane chemotherapeutics have been reported to have synergistic effects, decrease side systemic toxicity, sensitize resistant cells to drugs and decrease their biologically effective doses more than curcumin alone. Therefore curcumin analogues could be of benefit in patients with refractory or resistant disease; However there is a further need to evaluate their safety, pharmacokinetics and tolerability in clinical trials.

## References

[1] Estimated Cancer Incidence, Mortality and Prevalence Worldwide in 2012. www.globocan.iarc.fr.

[2] Varga D., Deniz M., Schwentner L., Wiesmüller L. (2013). Ovarian cancer: In search of better marker systems based on DNA repair defects. Int. J. Mol. Sci..

[3] Kyrgiou M., Salanti G., Pavlidis N., Paraskevaidis E., Ioannidis J.P. (2006). Survival benefits with diverse chemotherapy regimens for ovarian cancer: meta-analysis of multiple treatments. J. Natl. Cancer Inst..

[4] Rustin G.J., van der Burg M.E., Griffin C.L., Guthrie D., Lamont A., Jayson G.C., Kristensen G., Mediola C., Coens C., Qian W. (2010). Early versus delayed treatment of relapsed ovarian cancer (MRC OV05/EORTC 55955): A randomized trial. Lancet.

[5] Kumaran G.C., Jayson G.C., Clamp A.R. (2009). Antiangiogenic drugs in ovarian cancer. Br. J. Cancer.

[6] Sharma T., Dhingra R., Singh S., Sharma S., Tomar P., Malhotra M., Bhardwaj T.R. (2013). Afliberecept: A novel VEGF targeted agent to explore the future perspectives of anti-angiogenic theraphy for the treatment of multiple tumors. Mini-Rev. Med. Chem..

[7] Cannistra S.A., Matulonis U.A., Penson R.T., Hambleton J., Dupont J., Mackey H., Douglas J., Burger R.A., Armstrong D., Wenham R. (2007). Phase II study of bevacizumab in patients with platinum-resistant ovarian cancer or peritoneal serous cancer. J. Clin. Oncol..

[8] Burger R.A., Brady M.F., Bookman M.A., Fleming G.F., Monk B.J., Huang H., Mannel R.S., Homesley H.D., Fowler J., Greer B.E. (2011). Incorporation of bevacizumab in the primary treatment of ovarian cancer. N. Engl. J. Med..

[9] Freeman S. ICON-7 confirms first-line bevacizumab is beneficial. www.oncologypractice.com.

[10] Perren T.J., Swart A.M., Pfisterer J., Ledermann J.A., Pujade-Lauraine E., Kristensen G., Carey M.S., Beale P., Cervantes A., Kurzeder C. (2011). A phase 3 trial of bevacizumab in ovarian cancer. N. Engl. J. Med..

[11] Garcia A., Singh H. (2013). Bevacizumab and ovarian cancer. Ther. Adv. Med. Oncol..

[12] Du Bois A., Quinn M., Thigpen T., Vermorken J., Avall-Lundqvist E., Bookman M., Bowtell D., Brady M., Casado A., Cervantes A. (2005). 2004 Consensus statements on the management of ovarian cancer: Final document of the 3rd International Gynecologic Cancer Intergroup Ovarian Cancer Consensus Conference (GCIG OCCC 2004). Ann. Oncol..

[13] Heitz F., Harter P., Barinoff J., Beutel B., Kannisto P., Grabowski J.P., Heitz J., Kurzeder C., du Bois A. (2012). Bevacizumab in the treatment of ovarian cancer. Adv. Ther..

[14] Mangerich A., Bürkle A. (2011). How to kill tumor cells with inhibitors of poly(ADP-ribosyl)ation. Int. J. Cancer.

[15] Underhill C., Toulmonde M., Bonnefoi H. (2011). A review of PARP inhibitors: From bench to bedside. Ann. Oncol..

[16] Audeh M.W., Carmichael J., Penson R.T., Friedlander M., Powell B., Bell-McGuinn K.M., Scott C., Weitzel J.N., Oaknin A., Loman N. (2010). Oral poly(ADP-ribose) polymerase inhibitor olaparib in patients with *BRCA1* or *BRCA2* mutations and recurrent ovarian cancer: A proof-of-concept trial. Lancet.

[17] Chen Y., Zhang L., Hao Q. (2013). Olaparib: A promising PARP inhibitor in ovarian cancer theraphy. Arch. Gynecol. Obstet..

[18] Fong P.C., Yap T.A., Boss D.S., Carden C.P., Mergui-Roelvink M., Gourley C., de Greve J., Lubinski J., Shanley S., Messiou C. (2010). Poly(ADP)-ribose polymerase inhibition: Frequent durable responses in BRCA carrier ovarian cancer correlating with platinum-free interval. J. Clin. Oncol..

[19] Spannuth W.A., Sood A.K., Coleman R.L. (2010). Farletuzumab in epithelial ovarian carcinoma. Expert Opin. Biol. Ther..

[20] Gibbs D.D., Theti D.S., Wood N., Green M., Raynaud F., Valenti M., Forster M.D., Mitchell F., Bavetsias V., Henderson E. (2005). BGC 945, a novel tumor-selective thymidylate synthase inhibitor targeted to α-folate receptor-overexpressing tumors. Cancer Res..

[21] Vergote I., Calvert H., Kania M., Kaiser C., Zimmermann A.H., Sehouli J.A. (2009). A randomised, double-blind, phase II study of two doses of pemetrexed in the treatment of platinum-resistant, epithelial ovarian primary peritoneal cancer. Eur. J. Cancer.

[22] Naumann R.W., Coleman R.L., Burger R.A., Sausville E.A., Kutarska E., Ghamande S.A., Gabrail N.Y., Depasquale S.E., Nowara E., Gilbert L. (2013). PRECEDENT: A randomized phase II trial comparing EC145 and pegylated liposomal doxorubicin (PLD) in combination, *vs.* PLD alone, in subjects with platinum-resistant ovarian cancer. J. Clin. Oncol..

[23] Vlahov I.R., Santhapuram H.K., Kleindl P.J., Howard S.J., Stanford K.M., Leamon C.P. (2006). Design and regioselective synthesis of a new generation of target chemotherapeutics. Part 1: EC145, A folic acid conjugate of desacetylvinblastine monohydrazine. Bioorg. Med. Chem. Lett..

[24] Boivin M., Lane D., Piché A., Rancourt C. (2009). CA125 (MUC16) tumor antigen selectively modulates the sensitivity of ovarian cancer cells to genotoxic drug-induced apoptosis. Gynecol. Oncol..

[25] Chen Y., Clark S., Wong T., Chen Y., Chen Y., Dennis M.S., Luis E., Zhong F., Bheddah S., Koeppen H. (2007). Armed antibodies targeting the mucin repeats of the ovarian cancer antigen, MUC16, are highly efficacious in animal tumor models. Cancer Res..

[26] Chauhan S.C., Kumar D., Jaggi M. (2009). Mucins in ovarian cancer diagnosis and therapy. J. Ovarian Res..

[27] McQuarrie S., Mercer J., Syme A., Suresh M., Miller G. (2005). Preliminary results of nanopharmaceuticals used in the radioimmunotherapy of ovarian cancer. J. Pharm. Pharm. Sci..

[28] Hiss D. (2012). Optimizing molecular-targeted therapies in ovarian cancer: The renewed surge of interest in ovarian cancer biomarkers and cell signaling pathways. J. Oncol..

[29] Gupta S.C., Sung B., Kim J.H., Prasad S., Li S., Aggarwal B.B. (2013). Multitargetting by tumeric, the golden spice: From kitchen to clinic. Mol. Food Res..

[30] Esatbeyoglu T., Huebbe P., Ernst I.M., Chin D., Wagner A.E., Rimbach G. (2012). Curcumin—From molecule to biological function. Angew. Chem. Int. Ed..

[31] Lee K.H., Kim B.S., Keum K.S., Yu H.H., Kim Y.H., Chang B.S., Ra J.Y., Moon H.D., Seo B.R., Choi N.Y. (2011). Essential oil of *Curcuma longa* inhibits streptococcus mutants biofilm formation. J. Food Sci..

[32] Thiagarajan R., Manikandan R. (2013). Antioxidants and cataract. Free Radic. Res..

[33] Duvoix A., Blasius R., Delhalle S., Schnekenburger M., Morceau F., Henry E., Dicato M., Diederich M. (2005). Chemopreventive and therapeutic effects of curcumin. Cancer Lett..

[34] Niederau C., Gopfert E. (1999). The effect of cheliodonium-and turmeric root extract on upper abdominal pain due to functional disorders of the biliary system. Results from a placebo-controlled double-blind study. Med. Clin..

[35] Sidhu G.S., Mani H., Gaddipati J.P., Singh A.K., Seth P., Banaudha K.K., Patnaik G.K., Maheshwari R.K. (1999). Curcumin enhances wound healing in streptozotocin induced diabetic rats and genetically diabetic mice. Wound Repair Regen..

[36] Basnet P., Skalko-Basnet N. (2011). Curcumin: An anti-inflammatory molecule from a curry spice on the path to cancer treatment. Molecules.

[37] Shehzad A., Wahid F., Lee Y.S. (2010). Curcumin in cancer chemoprevention: Molecular targets, pharmacokinetics, bioavailability, and clinical trials. Arch. Pharm..

[38] EFSA Panel on Food Additives and Nutrient Sources added to Food (ANS) (2010). Scientific opinion on the reevaluation of curcumin (E 100) as a food additive. EFSA J..

[39] Lao C.D., Ruffin M.T., Normolle D., Heath D.D., Murray S.I., Bailey J.M., Boggs M.E., Crowell J., Rock C.L., Brenner D.E. (2006). Dose escalation of a curcuminoid formulation. BMC Complement. Altern. Med..

[40] Burgos-Morón E., Calderon-Montano J.M., Salvador J., Robles A., Lopez-Lazaro M. (2010). The dark side of curcumin. Int. J. Cancer.

[41] Vareed S.K., Kakarala M., Ruffin M.T., Crowell J.A., Normolle D.P., Djuric Z., Brenner D.E. (2008). Pharmacokinetics of curcumin conjugate metabolites in healthy human subjects. Cancer Epidemiol. Biomark. Prev..

[42] Cheng A.L, Hsu C.H., Lin J.K., Hsu M.M., Ho Y.F., Shen T.S., Ko J.Y., Lin J.T., Lin B.R., Ming-Shiang W. (2001). Phase I clinical trial of curcumin, a chemopreventive agent, in patients with high-risk or pre-malignant lesions. Anticancer Res..

[43] Sharma R.A., Euden S.A., Platton S.L., Cooke D.N., Shafayat A., Hewitt H.R., Marczylo T.H., Morgan B., Hemingway D., Plummer S.M. (2004). Phase I clinical trial of oral curcumin: Biomarkers of systemic activity and compliance. Clin. Cancer Res..

[44] Sandur S.K., Pandey M.K., Sung B., Ahn K.S., Murakami A., Sethi G., Limtrakul P., Badmaev V., Aggarwal B.B. (2007). Curcumin, demethoxycurcumin, bisdemethoxycurcumin, tetrahydrocurcumin and turmerones differentially regulate anti-inflammatory and anti-proliferative responses through a ROS-independent mechanism. Carcinogenesis.

[45] Hong J., Bose M., Ju J., Ryu J.H., Chen X., Sang S., Lee M.J., Yang C.S. (2004). Modulation of arachidonic acid metabolism by curcumin and related h-diketone derivatives: Effects on cytosolic phospholipase A(2), cyclooxygenases and 5-lipoxygenase. Carcinogenesis.

[46] Anand P., Kunnumakkara A.B., Newman R.A., Aggarwal B.B. (2007). Bioavailability of curcumin: Problems and promises. Mol. Pharm..

[47] Raju G.S.R., Pavitra E., Nagaraju G.P., Ramesh K., El-Rayes B.F., Yu J.S. (2014). Imaging and curcumin delivery in pancreatic cancer cell lines using PEGylated α-Gd_2_(MoO_4_)_3_ mesoporous particles. Dalton Trans..

[48] Mohanty C., Sahoo S.K. (2010). The *in vitro* stability and *in vivo* pharmacokinetics of curcumin prepared as an aqueous nanoparticulate formulation. Biomaterials.

[49] Terlikowska K., Witkowska A., Terlikowski S. (2014). Curcumin in chemoprevention of breast cancer. Postep. Hig. Med. Dosw..

[50] Lv J., Shao Q., Wang H., Shi H., Wang T., Gao W., Song B., Zheng G., Kong B., Qu X. (2013). Effects and mechanisms of curcumin and basil polysaccharide on the invasion of SKOV3 cells and dendritic cells. Mol. Med. Rep..

[51] Shi M., Cai Q., Yao L., Mao Y., Ming Y., Ouyang G. (2006). Antiproliferation and apoptosis induced by curcumin in human ovarian cancer cells. Cell. Biol. Int..

[52] Zheng L.D., Tong Q.S., Wu C.H. (2006). Growth inhibition and apoptosis inducing mechanisms of curcumin on human ovarian cancer cell line A2780. Chin. J. Integr. Med..

[53] Zheng L., Tong Q., Wu C. (2004). Growth-inhibitory effects of curcumin on ovary cancer cells and its mechanisms. J. Huazhong Univ. Sci. Technol..

[54] Pan W., Yang H., Cao C., Song X., Wallin B., Kivin R., Lu S., Hu G., Di W., Wan Y. (2008). AMPK mediates curcumin-induced cell death in CaOV3 ovarian cancer cells. Oncol. Rep..

[55] Montopoli M., Ragazzi E., Froldi G., Caparrotta L. (2009). Cell-cycle inhibition and apoptosis induced by curcumin and cisplatin or oxaliplatin in human ovarian carcinoma cells. Cell Prolif..

[56] Ji C., Cao C., Lu S., Kivlin R., Amaral A., Kouttab N., Yang H., Chu W., Bi Z., Di W. (2008). Curcumin attenuates EGF-induced AQP3 up-regulation and cell migration in human ovarian cancer cells. Cancer Chemother. Pharmacol..

[57] Saydmohammed M., Joseph D., Syed V. (2010). Curcumin suppresses constitutive activation of STAT-3 by up-regulating protein inhibitor of activated STAT-3 (PIAS-3) in ovarian and endometrial cancer cells. J. Cell. Biochem..

[58] Watson J.L., Greenshields A., Hill R., Hilchie A., Lee P.W., Giacomantonio C.A., Hoskin D.W. (2010). Curcumin-induced apoptosis in ovarian carcinoma cells is p53-independent and involves p38 mitogen-activated protein kinase activation and down-regulation of Bcl-2 and survivin expression and Akt signaling. Mol. Carcinog..

[59] Zhao S.-F., Zhang X., Zhang X.-J., Shi X.-Q., Yu Z.-J., Kan Q.-C. (2014). Induction of microRNA-9 mediates cytotoxicity of curcumin against SKOV3 ovarian cancer cells. Asian Pac. J. Cancer Prev..

[60] Seo J.H., Jeong K.J., Oha W.J., Sul H.J., Sohn J.S., Kim Y.K., Cho D.Y., Kang J.K., Park C.G., Lee H.Y. (2010). Lysophosphatidic acid induces STAT3 phosphorylation and ovarian cancer cell motility: Their inhibition by curcumin. Cancer Lett..

[61] Rath K.S., McCann G.A., Cohn D.E., Rivera B.K., Kuppusamy P., Karuppaiyah S. (2013). Safe and targeted anticancer therapy for ovarian cancer using a novel class of curcumin analogs. J. Ovarian Res..

[62] Kálai T., Kuppusamy M.L., Balog M., Selvendiran K., Rivera B.K., Kuppusamy P., Hideg K. (2011). Synthesis of *N*-substituted 3,5-bis(arylidene)-4-piperidones with high antitumor and antioxidant activity. J. Med. Chem..

[63] Selvendiran K., Ahmed S., Dayton A., Kuppusamy M.L., Tazi M., Bratasz A., Tong L., Rivera B.K., Kálai T., Hideg K. (2010). Safe and targeted anticancer efficacy of a novel class of antioxidant-conjugated difluoro-diarylidenylpiperidones: Differential cytotoxicity in healthy and cancer cells. Free Radic. Biol. Med..

[64] Dayton A., Selvendiran K., Kuppusamy M.L., Rivera B.K., Meduru S., Tamás K., Hideg K., Kuppusamy P. (2010). Cellular uptake, retention and bioabsorption of HO-3867, a fluorinated curcumin analog with potential antitumor properties. Cancer Biol. Ther..

[65] Samuni Y., Gamson J., Samuni A., Yamada K., Russo A., Krishna M.C., Mitchell J.B. (2004). Factors influencing nitroxide reduction and cytotoxicity *in vitro*. Antioxid. Redox Signal..

[66] Mitchell J.B., Krishna M.C., Kuppusamy P., Cook J.A., Russo A. (2001). Protection against oxidative stress by nitroxides. Exp. Biol. Med..

[67] Kuppusamy P., Li H., Ilangovan G., Cardounel A.J., Zweier J.L., Yamada K., Krishna M.C., Mitchell J.B. (2002). Noninvasive imaging of tumor redox status and its modification by tissue glutathione levels. Cancer Res..

[68] Kuppusamy P., Wang P., Shankar R.A., Ma L., Trimble C.E. (1998). *In vivo* topical EPR spectroscopy and imaging of nitroxide free radicals and polynitroxyl-albumin. Magn. Reson. Med..

[69] Selvendiran K., Tong L., Bratasz A., Kuppusamy M.L., Ahmed S., Ravi Y., Trigg N.J., Rivera B.K., Kálai T., Hideg K. (2010). Anticancer efficacy of a difluorodiarylidenyl piperidone (HO-3867) in human ovarian cancer cells and tumor xenografts. Mol. Cancer Ther..

[70] Selvendiran K., Ahmed S., Dayton A., Ravi Y., Kuppusamy M.L., Bratasz A., Rivera B.K., Kálai T., Hideg K., Kuppusamy P. (2010). HO-3867, a synthetic compound, inhibits migration and invasion of ovarian carcinoma cells through down-regulation of fatty acid synthase and focal adhesion kinase. Mol. Cancer Res..

[71] Selvendiran K., Ahmed S., Dayton A., Kuppusamy M.L., Rivera B.K., Kálai T., Hideg K., Kuppusamy P. (2011). HO-3867, a curcumin analog, sensitizes cisplatin-resistant ovarian carcinoma, leading to therapeutic synergy through STAT3 inhibition. Cancer Biol. Ther..

[72] Yallapu M.M., Gupta B.K., Jaggi M., Chauhan S.C. (2010). Fabrication of curcumin encapsulated PLGA nanoparticles for improved therapeutic effects in metastatic cancer cells. J. Colloid Interface Sci..

[73] Yallapu M.M., Maher D.M., Sundram V., Bell M.C., Jaggi M., Chauhan S.C. (2010). Curcumin induces chemo/radio-sensitization in ovarian cancer cells and curcumin nanoparticles inhibit ovarian cancer cell growth. J. Ovarian Res..

[74] Qu W., Xiao J., Zhang H., Chen Q., Wang Z., Shi H., Gong L., Chen J., Liu Y., Cao R. (2013). B19, a novel monocarbonyl analogue of curcumin, induces human ovarian cancer cell apoptosis via activation of endoplasmic reticulum stress and the autophagy signaling pathway. Int. J. Biol. Sci..

[75] Zhang X., Zhang H.Q., Zhu G.-H., Wang Y.-H., Yu X.-C., Zhu X.-B., Liang G., Xiao J., Li X.K. (2012). A novel mono-carbonyl analogue of curcumin induces apoptosis in ovarian carcinoma cells via endoplasmic reticulum stress and reactive oxygen species production. Mol. Med. Rep..

[76] Carew J.S., Nawrocki S.T., Cleveland J.L. (2007). Modulating autophagy for therapeutic benefit. Autophagy.

[77] Tang H., Murphy C.J., Zhang B., Shen Y., van Kirk E.A., Murdoch W.J., Radosz M. (2010). Curcumin polymers as anticancer conjugates. Biomaterials.

[78] Szakacs G., Annereau J.P., Lababidi S., Shankavaram U., Arciello A., Bussey K.J., Reinhold W., Guo Y., Kruh G.D., Reimers M. (2004). Predicting drug sensitivity and resistance: Profiling ABC transporter genes in cancer cells. Cancer Cell.

[79] Samimi G., Fink D., Varki N.M., Husain A., Hoskins W.J., Alberts D.S., Howell S.B. (2000). Analysis of MLH1 and MSH2 expression in ovarian cancer before and after platinum drug-based chemotherapy. Clin. Cancer Res..

[80] Boudsocq F., Benaim P., Canitrot Y., Knibiehler M., Ausseil F., Capp J.P., Bieth A., Long C., David B., Massiot G. (2005). Modulation of cellular response to cisplatin by a novel inhibitor of DNA polymerase β. Mol. Pharmacol..

[81] Canitrot Y., Cazaux C., Frechet M., Bouayadi K., Lesca C., Salles B., Hoffmann J.S. (1998). Overexpression of DNA polymerase β in cell results in a mutator phenotype and a decreased sensitivity to anticancer drugs. Proc. Natl. Acad. Sci. USA.

[82] Dabholkar M., Vionnet J., Bostick-Bruton F., Yu J.J., Reed E. (1994). Messenger RNA levels of XPAC and ERCC1 in ovarian cancer tissue correlate with response to platinum based chemotherapy. J. Clin. Investig..

[83] Helleman J., van Staveren I.L., Dinjens W.N., van Kuijk P.F., Ritstier K., Ewing P.C., van der Burg M.E., Stoter G., Berns E.M. (2006). Mismatch repair and treatment resistance in ovarian cancer. BMC Cancer.

[84] Swisher E.M., Sakai W., Karlan B.Y., Wurz K., Urban N., Taniguchi T. (2008). Secondary BRCA1 mutations in BRCA1-mutated ovarian carcinomas with platinum resistance. Cancer Res..

[85] Taniguchi T., Tischkowitz M., Ameziane N., Hodgson S.V., Mathew C.G., Joenje H., Mok S.C., D’Andrea A.D. (2003). Disruption of the Fanconi anemia-BRCA pathway in cisplatin-sensitive ovarian tumors. Nat. Med..

[86] Grompe M., D’Andrea A.D. (2001). Fanconi anemia and DNA repair. Hum. Mol. Genet..

[87] D’Andrea A.D., Grompe M. (2003). The Fanconi anemia/BRCA pathway. Nat. Rev. Cancer.

[88] Domchek S.M., Tang J., Stopfer J., Lilli D.R., Hamel N., Tischkowitz M., Monteiro A.N., Messick T.E., Powers J., Yonker A. (2013). Biallelic deleterious BRCA1 mutations in woman with early-onset ovarian cancer. Cancer Discov..

[89] Chirnomas D., Taniguchi T., de la Vega M., Vaidya A.P., Vasserman M., Hartman A.R., Kennedy R., Foster R., Mahoney J., Seiden M.V. (2006). Chemosensitization to cisplatin by inhibitors of the Fanconi anemia/BRCA pathway. Mol. Cancer Ther..

[90] Limtrakul P., Chearwae W., Shukla S., Phisalphong C., Ambudkar S.V. (2007). Modulation of function of three ABC drug transporters, P-glycoprotein (ABCB1), mitoxantrone resistance protein (ABCG2) and multidrug resistance protein 1 (ABCC1) by tetrahydrocurcumin, a major metabolite of curcumin. Mol. Cell. Biochem..

[91] Chearwae W., Wu C.P., Chu H.Y., Lee T.R., Ambudkar S.V., Limtrakul P. (2006). Curcuminoids purified from turmeric powder modulate the function of human multidrug resistance protein 1 (ABCC1). Cancer Chemother. Pharmacol..

[92] Ganta S., Amiji M. (2009). Coadministration of paclitaxel and curcumin in nanoemulsion formulations to overcome multidrug resistance in tumor cells. Mol. Pharm..

[93] Lin Y.G., Kunnumakkara A.B., Nair A., Merritt W.M., Han L.Y., Armaiz-Pena G.N., Kamat A.A., Spannuth W.A., Gershenson D.M., Lutgendorf S.K. (2007). Curcumin inhibits tumor growth and angiogenesis in ovarian carcinoma by targeting the nuclear factor-κB pathway. Clin. Cancer Res..

[94] Srivanias G., Harikrishna D., Mansoor A. (2010). Curcumin enhances oral bioavailability and anti-tumor therapeutic efficacy of paclitaxel upon administration in nanoemulsion formulation. J. Pharm. Sci..

[95] Weir N.M., Selvendiran K., Kutala V.K., Tong L., Vishwanath S., Rajaram M., Tridandapani S., Anant S., Kuppusamy P. (2007). Curcumin induces G2/M arrest and apoptosis in cisplatin-resistant human ovarian cancer cells by modulating Akt and p38 MAPK. Cancer Biol. Ther..

[96] Chan M.M., Fong D., Soprano K.J., Holmes W.F., Heverling H. (2002). Inhibition of growth and sensitization to cisplatin-mediated killing of ovarian cancer cells by polyphenolic chemopreventive agents. J. Cell. Physiol..

[97] Yunos N.M., Beale P., Yu J.Q., Huq F. (2011). Synergism from sequenced combinations of curcumin and epigallocatechin-3-gallate with cisplatin in the killing of human ovarian cancer cells. Anticancer Res..

[98] Ferrari E., Lazzari S., Marverti G., Pignedoli F., Spagnolo F., Saladini M. (2009). Synthesis, cytotoxic and combined cDDP activity of new stable curcumin derivatives. Bioorg. Med. Chem..

[99] Yunos N.M., Beale P., Yu J.Q., Huq F. (2011). Synergism from the combination of oxaliplatin with selected phytochemicals in human ovarian cancer cell lines. Anticancer Res..

[100] Dhillon N., Aggarwal B.B., Newman R.A., Wolff R.A., Kunnumakkara A.B., Abbruzzese J.L., Ng C.S., Badmaev V., Kurzrock R. (2008). Phase II trial of curcumin in patients with advanced pancreatic cancer. Clin. Cancer Res..

[101] Bayet-Robert M., Kwiatkowski F., Leheurteur M., Gachon F., Planchat E., Abrial C., Mouret-Reynier M.A., Durando X., Barthomeuf C., Chollet P. (2010). Phase I dose escalation trial of docetaxel plus curcumin in patients with advanced and metastatic breast cancer. Cancer. Biol. Ther..

[102] Garcea G., Berry D.P., Jones D.J., Singh R., Dennison A.R., Farmer P.B., Sharma R.A., Steward W.P., Gescher A.J. (2005). Consumption of the putative chemopreventive agent curcumin by cancer patients: Assessment of curcumin levels in the colorectum and their pharmacodynamic consequences. Cancer Epidemiol. Biomark. Prev..

[103] Irving G.R.B., Howells L.M., Sale S., Kralj-Hans I., Atkin W.S., Clark S.K., Britton R.G., Jones D.J., Scott E.N., Berry D.P. (2013). Prolonged biologically active colonic tissue levels of curcumin achieved after oral administration—A clinical pilot study including assessment of patient acceptability. Cancer Prev. Res..

[104] Sharma R.A., McLelland H.R., Hill K.A., Ireson C.R., Euden S.A., Manson M.M., Pirmohamed M., Marnett L.J., Gescher A.J., Steward W.P. (2001). Pharmacodynamic and pharmacokinetic study of oral Curcuma extract in patients with colorectal cancer. Clin. Cancer Res..

[105] Steward W.P., Gescher A.J. (2008). Curcumin in cancer management: Recent results of analogue design and clinical studies and desirable future research. Mol. Nutr. Food Res..

